# IoT and Satellite Sensor Data Integration for Assessment of Environmental Variables: A Case Study on NO_2_

**DOI:** 10.3390/s22155660

**Published:** 2022-07-28

**Authors:** Jernej Cukjati, Domen Mongus, Krista Rizman Žalik, Borut Žalik

**Affiliations:** Faculty of Electrical Engineering and Computer Science, University of Maribor, Koroška Cesta 46, SI-2000 Maribor, Slovenia; domen.mongus@um.si (D.M.); krista.zalik@um.si (K.R.Ž.); borut.zalik@um.si (B.Ž.)

**Keywords:** Internet of Things (IoT), remote sensing, sensor integration, machine learning, ensemble method

## Abstract

This paper introduces a novel approach to increase the spatiotemporal resolution of an arbitrary environmental variable. This is achieved by utilizing machine learning algorithms to construct a satellite-like image at any given time moment, based on the measurements from IoT sensors. The target variables are calculated by an ensemble of regression models. The observed area is gridded, and partitioned into Voronoi cells based on the IoT sensors, whose measurements are available at the considered time. The pixels in each cell have a separate regression model, and take into account the measurements of the central and neighboring IoT sensors. The proposed approach was used to assess NO${}_{2}$ data, which were obtained from the Sentinel-5 Precursor satellite and IoT ground sensors. The approach was tested with three different machine learning algorithms: 1-nearest neighbor, linear regression and a feed-forward neural network. The highest accuracy yield was from the prediction models built with the feed-forward neural network, with an RMSE of 15.49 $\times 10^{-6}$ mol/m${}^{2}$.

## 1. Introduction

Air pollution, global warming and other pollutants have a great impact on the environment, and have become a major global concern [1]. NO${}_{2}$, which is used as the case study in this paper, is one of the greenhouse gases, and an important indicator of air pollution. It is also a precursor for several harmful secondary air pollutants, such as ozone and particulate matter (PM${}_{2.5}$, and PM${}_{10}$). This was the reason why networks of in situ Internet of Things (IoT) sensors were established for monitoring environmental variables [2,3]. IoT sensors are low cost, easy to install and can perform measurements with high temporal resolution [4,5,6,7]. However, today, the networks of IoT sensors do not cover larger areas. On the other hand, space agencies have also addressed this issue by launching satellites equipped with instruments to observe air pollutants in the Earth’s atmosphere [8]. Satellite measurements assure large coverage, but their temporal resolution is low. Satellites provide measurements in the form of raster images, associated with various environmental attributes [9]. In this context, the spatiotemporal alignment of IoT and satellite data sources represents the main challenge of low-level data fusion approaches that can limit the efficiency of higher levels significantly. Past studies addressed the issue of spatiotemporal alignment, either by interpolation or simulation of monitored sensor values, to match the spatial resolution of satellite images, while feeding the aligned features into the higher level analytics tools [10].

In comparison to simulations, interpolation approaches are usually less computationally demanding. The most commonly used approaches include simple aggregation of the closest sensor values (i.e., Voronoi Natural Neighbors’ Interpolation), linear and bilinear interpolation, Inverse Distance Weighting (IDW) and kriging [11,12]. The aggregations of the closest sensor values are the most straightforward, as they do not require any additional data processing for estimating target values, such as, for example, the mixed effect regression model of daily ground NO${}_{2}$ concentrations from Aura satellite measurements, demographic and thematic maps (e.g., roads and elevations), as well as aggregation of sensor data from the nearest weather station to the given location were examined in [13]. More recently, Zhan et al. [14] estimated daily NO${}_{2}$ concentrations by additionally considering the daily Planetary Boundary Layer Height (PBLH) and Normalized Difference Vegetation Index (NDVI), while applying co-kriging to interpolate the meteorological data. Alternatively, spatial data alignment of raster data was achieved using interpolation with area weighted averages, while temporal convolution with Gaussian kernels was used to fill the missing values within the satellite images. The interpolated values were used in the combination of the random forest and the spatiotemporal kriging to estimate the daily pollutant exposure. Improved accuracy, however, was reported by using bilinear interpolation for increasing the resolution of the satellite data. IDW was used for interpolation of missing satellite values, together with kriging-based interpolation of meteorological sensors’ data fed into XGBoost regression [15]. Alternatively, Araki et al. [16] used Aura satellite measurements to estimate monthly ground NO${}_{2}$ concentrations. The data of roads, demography, land use and positions of large combustion sources were considered, in addition to meteorological data. While the satellite data were re-gridded using bilinear interpolation, ordinary kriging was utilized to grid the values of meteorological sensor’s data. The study proposed the combination of land use regression with the random forest to estimate the target variables. They confirmed that Land-Use Random Forest performs better than land-use regression. Nevertheless, interpolation approaches inevitably introduce inaccuracies into the definition of explanatory variables by neglecting the spatially-dependent variance in their behavior. As these may accumulate within the resulting NO${}_{2}$ data layer [10], significant effort was dedicated to the simulation-based approaches.

The numerical-based Chemical Transport Models (CTMs) are the most common amongst different simulation models [17]. They are employed to simulate atmospheric chemistry by dividing the atmosphere into grid cells and defining the behavior of chemical species of interest within them using a numerical model. The behavior of concentration levels may be dependent on various environmental parameters (e.g., wind direction, temperature or humidity), as well as on the characteristic of the considered pollutant [18]. Amongst many CTMs, the Goddard Earth Observing System–Chem (GEOS-Chem) and Weather Research and Forecasting (WRF) are the most popular [19] when considering the fusion of the meteorological sensors’ data. For example, Li et al. [20] estimated ground NO${}_{2}$ concentration levels using GEOS-Chem simulations of meteorological variables and nitric acid surface mass concentrations. They fed the raster layers with NO${}_{2}$ Sentinel-5 Precursor (Sentinel-5P) data, NDVI from Terra and Aqua data and a digital elevation model to a geographically and temporally weighted generalized regression neural network. Alternatively, Qin et al. [21] used the same regression model for an estimation of the ground level NO${}_{2}$ concentrations, based on the simulated meteorological parameters from WRF, together with Aura satellite NO${}_{2}$ retrievals and the interpolated population data. Recent studies also examined the usage of these models for simulating the behavior of target variables directly. Beloconi and Vounatsou [22] examined daily NO${}_{2}$ estimation using GEOS-Chem. Here, the simulation model was constructed to simulate the vertical distribution of NO${}_{2}$ based on retrievals from the Aura satellite. The results were then improved additionally by a Bayesian geostatistical regression model using a variety of other predictors, including land cover, tree cover density, terrain elevation, night-time lights, land surface temperature both day and night, NDVI, data of roads, and meteorological data. Similarly, Yang et al. [23] applied regression on the results of a simulation model for estimations of ground NO${}_{2}$ levels. Here, retrievals of NO${}_{2}$ from the Aura satellites were combined with Aerosol Optical Depth data from the Terra, Aqua, OrbView-2, and CALIPSO satellites within the GEOS-Chem model, while meteorological data were simulated by WRF. Additional predictor variables feeding the supervised forward stepwise linear regression model included position, land use variables, traffic, and wind data. Other types of models and their combinations were studied (e.g., Community Multiscale Air Quality and GEOS-Chem) with a variety of regressions (neural network, random forest, gradient boosting algorithms and a generalized additive geographically weighted model) [24]. However, CTM-based simulation approaches are computationally demanding and difficult to implement, as they require precise definition of the behavior of chemical species within the given grid-cell. Consequently, the results are of low spatial and temporal resolutions [25].

The methods mentioned above perform interpolation in the pre-processing step to fill the missing spatial gaps. The proposed method omits interpolation during the initialization. Instead, the assessment of an environmental variable is performed by an ensemble of regression models, where each regression model performs the interpolation by different parameters.

The whole process constructs a satellite-like raster image based on the in situ IoT measurements. The resulting image can, therefore, be constructed in times when IoT measurements are given in comparison with other methods, which expose lower temporal resolutions (typically on the dally, or even monthly, scale). For this, the observed area is partitioned into Voronoi cells, based on the locations of the IoT sensors active at the desired time. Each set of pixels located in a Voronoi cell has its own regression model, by which better adaptation can be achieved to the local characteristics. The parameters for the regression models are selected by using the measurements of the neighboring IoT sensors. The nearest neighbor, linear regression, and forward-feeding neural network are used in this paper. Accordingly, the proposed approach brings the following novelties:

- a strong theoretical foundation for modeling the relationship between the IoT and satellite data,
- the integration of interpolation directly into regression models, yielding a more compact and consistent algorithm,
- an ensemble of base regression models constructed by using measurements from the surrounding IoT sensors, and
- an increased temporal resolution, dependent only on the sampling rate of the IoT sensors.

The rest of the paper is organized as follows: The details of the proposed method are explained in Section 2. Section 3 describes the observed area and data preparation. Section 4 provides the results of the approach and their evaluation. Section 5 discusses the obtained results and concludes the article.

## 2. Methods

**Figure 1:**
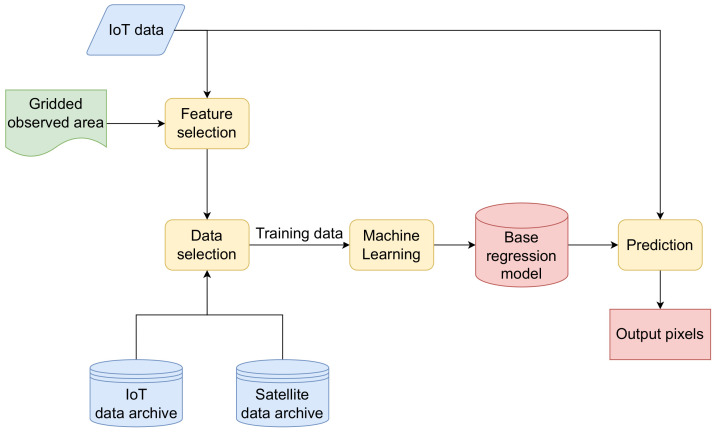
The architecture of the proposed method.

This Section introduces a new ensemble method for assessing the environmental variables by constructing a satellite-like image using the data fusion paradigm. The method’s input consists of the gridded observed area, in situ IoT sensor data, and the two archives. The first archive contains satellite images and the second archive stores the past IoT measurements. To construct the satellite-like image, the following steps are implemented (see Figure 1):

- Feature selection,
- Data selection, and
- Machine learning and Prediction.

These steps are highlighted in the continuation.

### 2.1. Feature Selection

**Figure 2:**
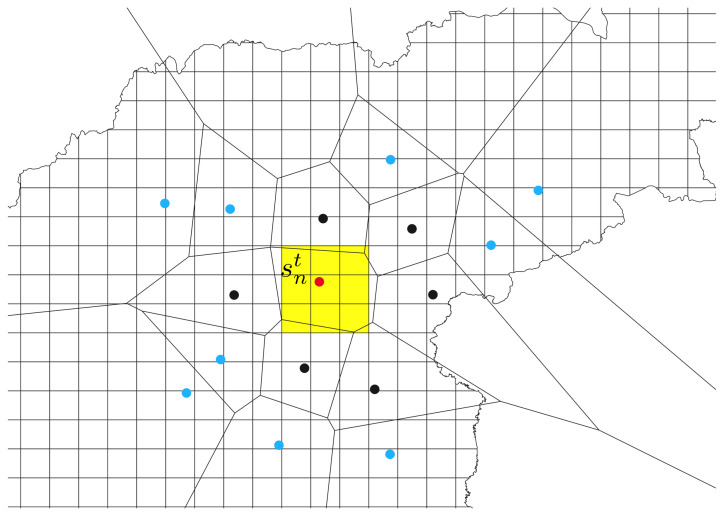
Voronoi diagram constructed on the sensors, where sensor $s_{n}^{t}$ is marked by the red dot, its natural neighbors by the black dots, while the remaining sensors are blue. Pixels whose centers are located within the Voronoi cell defined by $s_{n}^{t}$, represent target pixels and are colored in yellow. The polyline symbolizes the border of the observed area.

Let $S=\{s_{n}\},0\leq n<N$, be a set of *N* IoT sensors. Each sensor $s_{n}$ operates at the fixed location *Position($s_{n}$)* = ( $x_{n}$, $y_{n}$), and can either be available or turned off. $S^{t}$= {$s_{n}^{t}$}, $S^{t}\subseteq S$ denotes a set of these sensors, which are returning the valid measurements of an environmental variable *E* in the given time *t*$\in [\;\;t_{s},t_{e}]\;\;$, where $t_{s}$ indicates the starting and $t_{e}$ the ending time of the measurements. As seen in Figure 2, the observed area is covered by a set of *M* pixels $P=\{p_{m}\}$, where $p_{m}$ has the center at the location $Position\left(p_{m}\right)=\left(\;\;x_{m},y_{m}\right)\;\;,\;0\leq m<M$.

Let $V=\{v_{n}\}$ be a set of *N* Voronoi cells. To select the appropriate features for the machine learning, the observed area is partitioned into a set of Voronoi cells $V^{t}=\left\{v_{n}^{t}\right\}$, $V^{t}\subseteq V$, in given time *t*, where each active IoT sensor $s_{n}^{t}$ represents the Voronoi center of the Voronoi cell $v_{n}^{t}$. The neighboring Voronoi cells (and the neighbors of sensor $s_{n}^{t}$) are then determined for each Voronoi cell $v_{n}^{t}$: $\Gamma \left(v_{n}^{t}\right)=\left\{v_{i}^{t}\right\}$ and $\Gamma \left(s_{n}^{t}\right)=\left\{s_{i}^{t}\right\}$; $s_{i}^{t}\subseteq S^{t}$. Sensors $s_{n}^{t}$ and $\Gamma (s_{n}^{t})$ are then contained in a vector of the selected sensors $F^{t}$ at the time moment *t*, $F^{t}=s_{n}^{t}\cup \Gamma \left(\;\;s_{n}^{t}\right)\;\;$, which is then used in the data selection process.

### 2.2. Data Selection

By utilizing knowledge about the past measurements of the observed environmental variable *E* by sensors and by satellite images, the regression models are built for each vector of the target pixels $\mathrm{TP}(v_{n}^{t})$, which are located inside the Voronoi cell $v_{n}^{t}$, $\mathrm{TP}\left(v_{n}^{t}\right)=Inside\left(v_{n}^{t}\right),\mathrm{TP}\left(v_{n}^{t}\right)\subseteq P$ (see Figure 2). The measurements from both sources are stored as time series in two archives: Archive $A_{\mathrm{IoT}}$ containing times and measurements of E from *N* IoT sensors $A_{\mathrm{IoT}}=\left\{Time,E\_s_{0},E\_s_{1},...,E\_s_{N-1}\right\}$, while $A_{\mathrm{Sat}}=\left\{Time,E\_p_{0},E\_p_{1},...,E\_p_{M-1}\right\}$ stores the measurements of *E* obtained from *M* pixels of the satellite images. Possible invalid values in $A_{\mathrm{IoT}}$ are identified and marked. The obtained satellite images are re-gridded, due to inconsistencies in the pixel position in each revisited time [16] before being stored in $A_{\mathrm{Sat}}$. In addition, for each m−th pixel $p_{m}$, its validity value is also stored beside the value of *E*.

**Figure 3:**
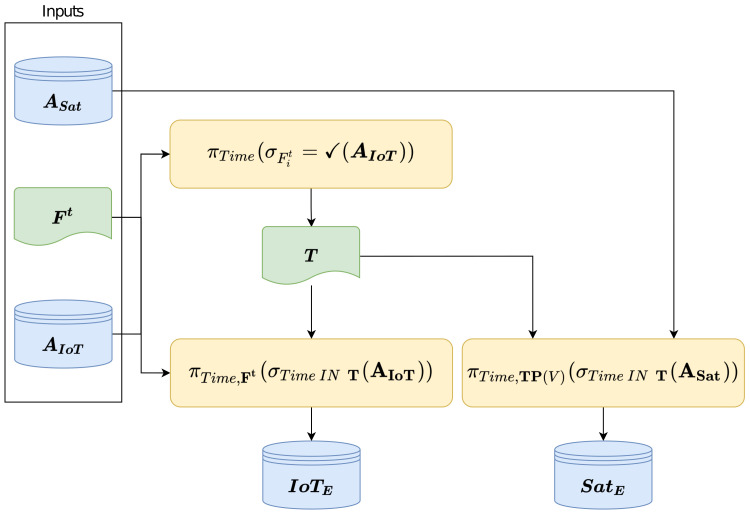
Data selection process.

The selection process (see Figure 3) consists of querying both archives using $F^{t}$. All time moments, where values from IoT sensors are valid, are used and stored in vector **T**. To construct the training, validation and testing datasets, the vector **T** is used to obtain Tables ${\mathrm{IoT}}_{E}$ and ${\mathrm{Sat}}_{E}$, which are aligned temporally.

#### Data Selection in Details

In the continuation, the whole selection process is described in detail, with the relation algebra operators’ projection (π) and selection (σ).

Archive $A_{\mathrm{IoT}}$ is queried firstly by a vector of the selected sensors from vector $F^{t}$ obtained during the feature selection process. The query’s result is a vector of times $T,T\subseteq [\;\;t_{s},t_{e}]\;\;$, (Equation (1)), where ✓ denotes valid measurements.

(1) $$T=\pi_{Time}\left(\;\sigma_{F_{i}^{t}=✓}\left(\;A_{\mathrm{IoT}}\right)\;\right)\;,0\leq i<\left|F^{t}\right|$$

A matrix ${\mathrm{IoT}}_{E}$, of available measurements of *E* obtained by IoT sensors in times **T**, is then acquired by querying the archive $A_{\mathrm{IoT}}$ (Equation (2)).

(2) $${\mathrm{IoT}}_{E}=\pi_{Time,F^{t}}\left(\;\sigma_{Time\;IN\;T}\left(\;A_{\mathrm{IoT}}\right)\;\right)\;$$

Subsequently, the archive $A_{\mathrm{Sat}}$ (Equation (3)) is queried for measurements of *E* from satellite pixels (${\mathrm{Sat}}_{E}$), for time moments **T** obtained from (Equation (1)), and target pixels $\mathrm{TP}(v_{n}^{t})$, where $v_{n}^{t}$ is the Voronoi cell of sensor $s_{n}^{t}$.

(3) $${\mathrm{Sat}}_{E}=\pi_{Time,\mathrm{TP}(v_{n}^{t})}\left(\;\sigma_{Time\;IN\;T}\left(\;A_{\mathrm{Sat}}\right)\;\right)\;$$

**Figure 4:**
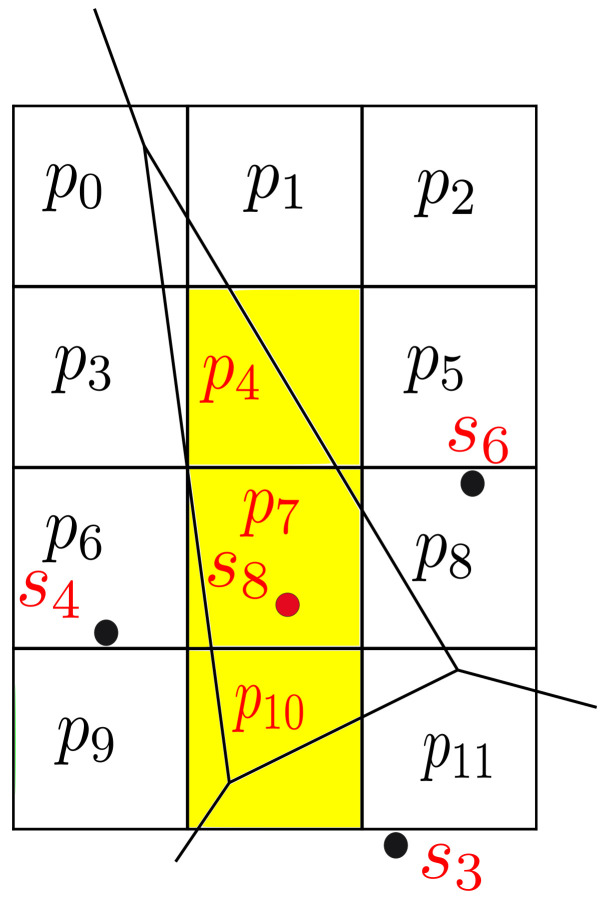
Voronoi diagram for the example presented in Table 1 and Table 2. The center of the Voronoi cell is marked by red dot, while its natural neighbors are marked by black dots. Pixels whose centers are located within the Voronoi cell are colored in yellow. The extraction from the whole observed area is shown, where the pixels from $p_{0}$ to $p_{11}$ are present.

A concrete example is demonstrated in Table 1 and Table 2, where the valid measurements are represented schematically by tick marks, while the invalid ones are crossed. The feature selected sensors $F^{t}=\left\{s_{3},s_{4},s_{6},s_{8}\right\}$ for sensor $s_{8}$ and $\Gamma \left(s_{8}\right)=\left\{s_{3},s_{4},s_{6}\right\}$ are seen in Figure 4.

The $A_{\mathrm{IoT}}$ is queried (Equation (4)).

(4) $$T=\pi_{Time}\left(\;\sigma_{E\_s_{3}=✓AND\;\;E\_s_{4}=✓AND\;\;E\_s_{6}=✓AND\;\;E\_s_{8}=✓}\left(\;A_{\mathrm{IoT}}\right)\;\right)\;$$

The query’s result is vector $T=\{t_{3},t_{4},t_{6},t_{7}\}$. The ${\mathrm{IoT}}_{E}$ is then obtained (Equation (5)).

(5) $${\mathrm{IoT}}_{E}=\pi_{Time,E\_s_{3},E\_s_{4},E\_s_{6},E\_s_{8}}\left(\;\sigma_{Time\;IN\;T}\left(\;A_{\mathrm{IoT}}\right)\;\right)\;$$

Similarly, let us assume that sensor $s_{8}$ (located in Voronoi cell $v_{8}$ in Figure 4) has the following 3 target pixels: $\mathrm{TP}\left(\;v_{8}\right)\;=\left\{p_{4},p_{7},p_{10}\right\}$. The $A_{\mathrm{Sat}}$ is queried and ${\mathrm{Sat}}_{E}$ is obtained (Equation (6)).

(6) $${\mathrm{Sat}}_{E}=\pi_{Time,E\_p_{4},E\_p_{7},E\_p_{10}}\left(\;\sigma_{Time\;IN\;T}\left(\;A_{\mathrm{Sat}}\right)\;\right)\;$$

The results of queries from both archives are the yellow-colored cells in Table 1 and Table 2.

### 2.3. Machine Learning and Prediction

The base regression models for satellite-like image construction were obtained by three different approaches:

- Nearest Neighbor,
- Linear Regression, and
- Neural Networks.

The same data from ${\mathrm{IoT}}_{E}$, ${\mathrm{Sat}}_{E}$ and the input feature vector ${\mathrm{FV}}^{t}=<F^{t},E\_F^{t}>$ are used by all three approaches. Let us remember that $F^{t},F^{t}=s_{n}^{t}\cup \Gamma \left(\;s_{n}^{t}\right)\;\;$, while $E\_F^{t}$ represents their measurements of the observed environmental variable *E*, $E\_F^{t}=E\_s_{n}^{t}\cup E\_\Gamma \left(\;s_{n}^{t}\right)\;\;$.

#### 2.3.1. Nearest Neighbor

Nearest Neighbor (1-NN) is an instance-based machine learning algorithm [26]. It queries the training dataset of instances to find the nearest object. In our case, 1-NN is used to find the nearest instance from ${\mathrm{IoT}}_{E}$ according to input feature vector ${\mathrm{FV}}^{t}=<F^{t},E\_F^{t}>$ by which the corresponding values are obtained from ${\mathrm{Sat}}_{E}$. The distance *D* between ${\mathrm{FV}}^{t}$ and instance in the *row* from ${\mathrm{IoT}}_{E},{\mathrm{IoT}}_{E,\mathrm{row}},0\leq \mathrm{row}<\left|{\mathrm{IoT}}_{E}\right|$, is calculated by Equation (7). The distance *D* is the sum of differences between the measurements of $E\_F^{t}$ from vector ${\mathrm{FV}}^{t}$, multiplied with the Inverse Distance Weighting (*IDW*) between the central sensor $s_{n}^{t}$ and each of its neighbors in $\Gamma (\;s_{n}^{t})$. Due to being the result of $\mathrm{IDW}(\;s_{n}^{t},s_{n}^{t})\;=1$, this is omitted, and only the difference between $E\_s_{n}^{t}$ and $IoT_{E,\mathrm{row}}\_s_{n}$ is made at the start of calculation.

(7) $$\begin{array}{cc} D(\;\;{\mathrm{FV}}^{t},{\mathrm{IoT}}_{E,\mathrm{row}})\; & =|E\_s_{n}^{t}-IoT_{E,\mathrm{row}}\_s_{n}|\\ \; & +\sum_{column=0}^{|\Gamma (\;s_{n}^{t})\;|-1}\mathrm{IDW}\left(\;s_{n}^{t},\Gamma {\left(\;s_{n}^{t}\right)}_{column}\right)\;\times \left|E\_\Gamma {\left(\;s_{n}^{t}\right)}_{column}-IoT_{E}\_\Gamma {\left(\;s_{n}^{t}\right)}_{row,column}\right| \end{array}$$

The $E\_s_{n}^{t}$ in Equation (7) denotes the measurements of *E* in time moment *t*, $E\_s_{n}^{t}\in E\_F^{t}$, while ${\mathrm{IoT}}_{E,\mathrm{row}}\_s_{n}$ is sensor $s_{n}$’s measurements from ${\mathrm{IoT}}_{E,\mathrm{row}}$ (Equation (8)).

(8) $$IoT_{E,\mathrm{row}}\_s_{n}=\pi_{E\_s_{n}}\left(\;{\mathrm{IoT}}_{E,\mathrm{row}}\right)\;$$

*IDW* is calculated by Equation (9).

(9) $$\mathrm{IDW}\left(\;s_{n}^{t},\Gamma {\left(\;s_{n}^{t}\right)}_{column}\right)\;=\frac{1}{\sqrt{\mathrm{Position}\left(\;s_{n}^{t}\right)\;{\;}^{2}+\mathrm{Position}\left(\;\;\Gamma \left(\;s_{n}^{t}\right){\;}_{column}\right)\;{\;}^{2}}}$$

Furthermore, the measurements of E from ${\mathrm{IoT}}_{E}\_\Gamma {\left(\;s_{n}^{t}\right)}_{\mathrm{row}}$ are obtained by Equation (10).

(10) $${\mathrm{IoT}}_{E}\_\Gamma \left(\;s_{n}^{t}\right)\;{\;}_{\mathrm{row}}=\pi_{E}\left(\;\sigma_{E\_s\;IN\;\Gamma (\;s_{n}^{t})\;}\left(\;{\mathrm{IoT}}_{E},{\;}_{\mathrm{row}}\right)\;\right)\;$$

The values of E of the target pixels $\mathrm{TP}(\;v_{n}^{t})\;$, which are obtained from the satellite, are denoted as $E\_\mathrm{TP}(\;v_{n}^{t})\;\;$. They are obtained from a *row* in Table ${\mathrm{Sat}}_{E}$. The index *row* is acquired by applying the *Argmin* function, which returns the index in which *D* is minimal (Equation (11)).

(11) $$E\_\mathrm{TP}\left(\;v_{n}^{t}\right)\;={\mathrm{Sat}}_{E,\mathrm{row}}=\mathrm{Argmin}\left(\;\;D\left(\;{\mathrm{FV}}^{t},{\mathrm{IoT}}_{E,\mathrm{row}}\right)\;\right)\;$$

The pixels from $\mathrm{TP}(\;v_{n}^{t})\;$ may contain some invalid values. For such pixels the valid values are used from the next nearest instance $\mathrm{row}’=\mathrm{Argmin}(\;D\left(\;{\mathrm{FV}}^{t},{\mathrm{IoT}}_{E,\mathrm{row}}\right)\;)\;\;$. The process is repeated until all $E\_\mathrm{TP}(\;v_{n}^{t})\;$ are valid.

#### 2.3.2. Linear Regression

Linear Regression (LR) also takes advantage of supervised learning to model the relationships between variables [27]. LR is used to model the relationship between the measurements of E from the IoT sensors and a single value of each target pixel from the satellite, $E\_F^{t}\mapsto E\_p_{i},0\leq i<\left|E\_\mathrm{TP}\left(\;v_{n}^{t}\right)\;\;\right|$. Consequently, the algorithm uses only the values from ${\mathrm{IoT}}_{E}$ to calculate the value of the targeted pixel $E\_p_{i}$. However, the samples with invalid pixel values are excluded. The result is a regression equation, which is represented by a vector of coefficients **b** (Equation (12)).

(12) $$E\_p_{i}=b_{0}+\sum_{l=0}^{|F^{t}|-1}b_{l+1}E\_F_{l}^{t}$$

#### 2.3.3. Neural Networks

A Neural Network (NN) utilizes supervised learning [28]. The NN uses samples in the training dataset to generate a regression function that maps the inputs into their corresponding outputs. Our method uses the feed-forward neural network [29] to map the measurements of any environmental variable *E* from the IoT sensors to the values of the targeted pixels. The network consists of an input layer, a hidden layer, and an output layer. These layers are connected densely, meaning that each neuron of the current layer is connected to each one in the preceding layer. The values in each j–th layer are denoted as $x_{j,k}$, where j,0≤j<3 is the number of layers and k,0≤k<LayerSize(j) is the number of elements in each layer. The number of neurons in the hidden layer is the mean of the neurons in the input and output layers (Equation (13)).

(13) $$LayerSize\left(1\right)=\frac{LayerSize(0)+LayerSize(2)}{2}$$

The regression function $E\_F^{t}\mapsto E\_\mathrm{TP}\left(\;v_{n}^{t}\right)\;$ is generated in the process of machine learning. This is achieved by feeding the input samples from ${\mathrm{IoT}}_{E}$ to the NN and comparing the obtained output with samples from the ${\mathrm{Sat}}_{E}$. The initial values of $E\_F^{t}$ (also $x_{0}$) are then used as input to the neurons in the hidden layer. The input to neuron $ne_{j,k}$ is a sum of the input values $x_{j-1}$ multiplied by the vector of weights $w_{j,k}$ (Equation (14)).

(14) $$\mathrm{Input}\left(\;ne_{j,k}\right)\;=\sum_{k0=0}^{LayerSize(j-1)}w_{j,k,k0}\times x_{j,k0}$$

The activation function Φ is applied on the vector of the obtained values, where function *Softmax* [30] is applied in the hidden layer (Equation (15)), and, a linear activation function is used at the output layer.

(15) $$x_{j}=\Phi \left(\;\mathrm{Input}\left(\;{\mathrm{ne}}_{j}\right)\;\right)\;=\frac{e^{Input(\;ne_{j,k})\;}}{\sum_{k0=0}^{LayerSize(j-1)}e^{Input(\;ne_{j,k_{0}})\;}}$$

The weights of the regression function are then updated according to the calculated error between the actual value $E\_p_{i},E\_p_{i}\in E\_\mathrm{TP}\left(v_{n}^{t}\right)$ and the obtained value, which is denoted as $E\_p_{i}^{\prime }$. In the ${\mathrm{Sat}}_{E}$ some training samples can contain invalid measurements. For this purpose, the error is calculated according to Equation (16).

(16) $$\mathrm{error}=\left\{\begin{array}{cc} 0 & if\;E\_p_{i}\;is\;invalid\\ |E\_p_{i}-E\_p_{i}^{\prime }| & otherwise \end{array}\right.$$

## 3. Study Area and Data Preparation

**Figure 5:**
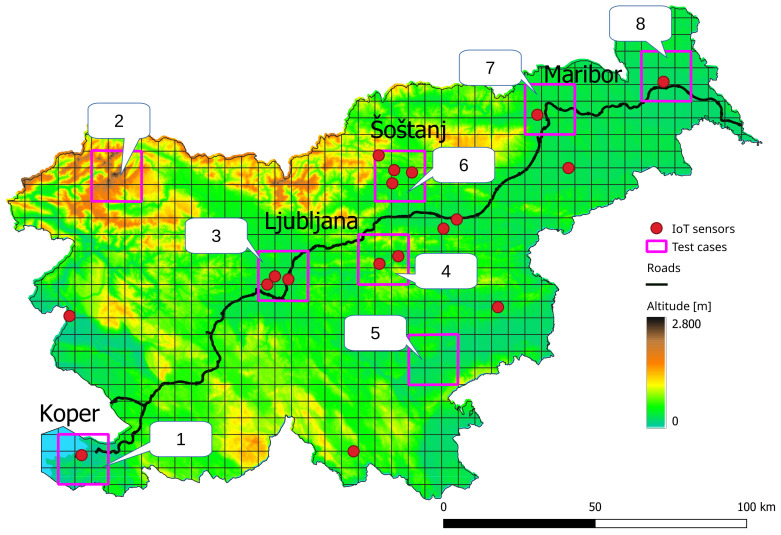
Location of the IoT ground sensors for NO${}_{2}$ and the eight testing cases.

In order to account for various testing conditions, the area of the Republic of Slovenia was used as the observed area. The country covers 20,271 square kilometers, and is known for its geomorphological diversity. This is due mostly to various natural regions gathered in one place: Alps, Dinaric Alps, the Pannonian Basin and the Mediterranean Basin. Although the area is mostly covered by forests (up to almost 60%), it bears a variety of pollution sources [31,32]. As seen in Figure 5, the area is covered sparsely by IoT sensors to monitor NO${}_{2}$ emissions. One of the main pollution sources is traffic, as roads which are part of Trans-European Transport Network, connecting major European and Slovenian cities, cross the country. Namely, Ljubljana and Maribor are places in Slovenia where the population is the most dense [33]. Furthermore, another polluted area connected to the road traffic is the port of Koper, which is an international connection from Continental Europe to the Mediterranean Sea [34,35]. Moreover, Slovenia owns the Šoštanj Thermal Power Plant located in the Šalek Valey, which is a large source of air pollution, as it produces approximately 35% of the electricity [36]. To obtain different testing scenarios, eight different areas across the whole country were selected as test cases.

Sentinel-5P is dedicated to the monitoring of the Earth’s atmosphere, and has a revisit rate of less than a day [37]. The measurements are given to users in the form of Network Common Data Form (format NetCDF) [38]. Each pixel is equipped with a timestamp, the value of the tropospheric NO${}_{2}$ column, and a quality assurance value (qa_value∈[0,1]), which indicates the validity of the measurement [37]. qa_value=0 corresponds to an invalid measurement, while qa_value=1 represents a value with no errors. Pixels with qa_value>0.75 were used as recommended in [39]. Due to the inconsistencies in the satellite pixel position at each revisit time, the observed area was gridded, as seen in Figure 5. Based on the typical satellite pixel sizes, the size 5.5×5.5 km was selected for the pixels in the grid. The satellite image was cropped and re-gridded to match the observed gridded area. Additionally, the data were used from the IoT ground sensors measuring NO${}_{2}$ seen in Figure 5. The unified interface providing various geo-biophysical parameters from sensor networks across Slovenia is available in [40]. The national data provider is the Slovenian Environment Agency [3]. However, other local providers contribute their data to the mentioned platform, such as, for example, the Šoštanj Thermal Power Plant.

## 4. Results

The proposed approach was implemented on a personal computer with an $Intel^{®}\;Core^{TM}i7-9750H$ CPU, 6 cores, 12 MB of cache and 32 GB of memory. The approach was tested with all three introduced machine learning algorithms.

The implementation of LR was taken from the programming library MLPACK [41]. Furthermore, the feed-forward neural network was implemented using TensorFlow [42]. The regression model was built by setting the hyperparameters: *epochs* to 250, *batch_size* to 16, and *optimizer* to *Adam*. These hyperparameters, along with the selection of the activation function and the number of neurons in the hidden layer, were obtained by using cross-validation with the Grid Search algorithm [43]. The parameter *cv*, which determines the number of folds was set to three. Additionally, the input data were scaled to a range between 0 and 1. On the other hand, the mean value was subtracted from the target variables in the training dataset. Additionally, they were divided by their Standard Deviation. The calculated outputs were then mapped back to their original range.

**Figure 6:**
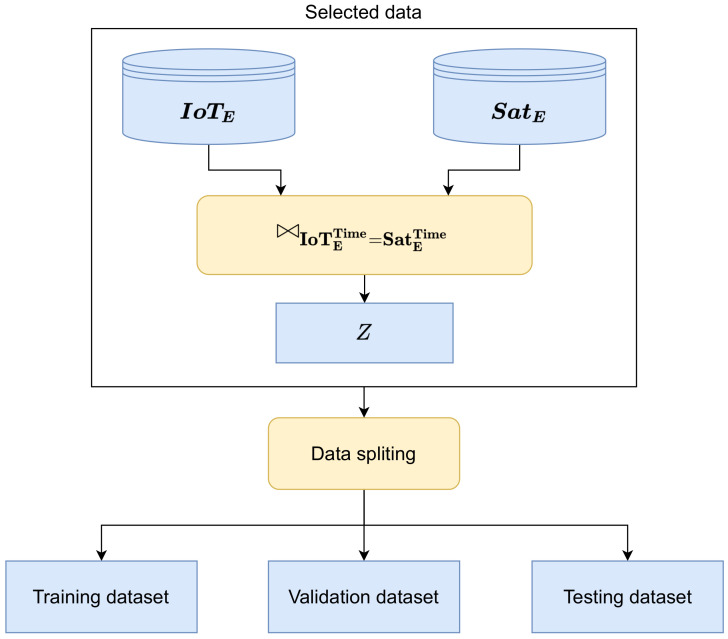
Data splitting into the training and testing datasets.

The Tables ${\mathrm{IoT}}_{E}$ and ${\mathrm{Sat}}_{E}$ were aligned temporally, and aggregated by applying the natural join ${⋈}_{conditions}$ (Equation (17)). The aggregated dataset was split in order to obtain the training, validation and testing datasets (see Figure 6).

(17) $$Z={⋈}_{{\mathrm{IoT}}_{E}^{\mathrm{Time}}={\mathrm{Sat}}_{E}^{\mathrm{Time}}}$$

**Figure 7:**
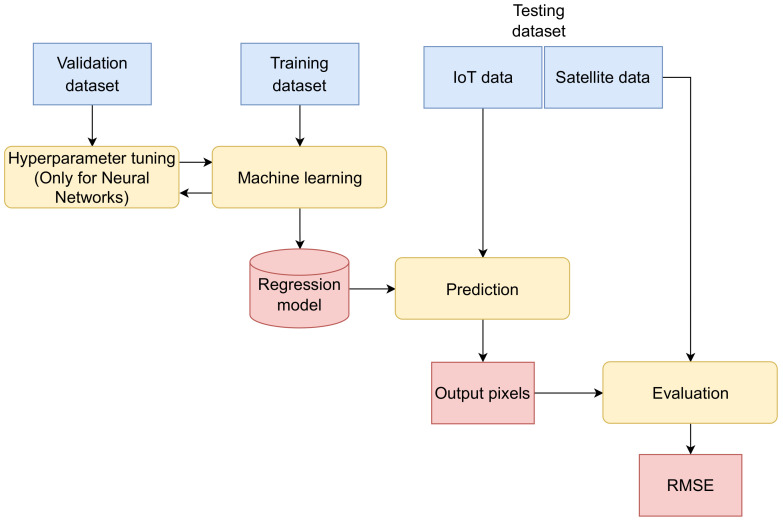
Machine learning and model evaluation workflow.

The training and validation datasets contained the samples between 1 January 2020 and 28 February 2021. As seen in Figure 7, the training dataset was used to build the prediction model [44]. The validation dataset was applied only during the tuning of the hyperparameters for the regression model built by the NN.

The testing dataset included the measurements from 1 March 2021, and up to and including 1 June 2021. This dataset was withheld from the machine learning, and was used to evaluate the regression model’s performance, as performed in [28,45].

The evaluation of the machine learning model was performed by comparing the calculated values and the actual measurements of *E* (i.e., NO${}_{2}$) from the testing dataset using the Root Mean Square Error (RMSE) metric, defined in Equation (18). The testing dataset included a total of 72,813 valid *E* values from the satellite pixels in P. Let us remember that P is a set of *M* pixels in the observed area, $P=\{p_{m}\},0\leq m<M$

(18) $$\mathrm{RMSE}=\sqrt{\frac{\sum_{p_{i}\in P}\left(\;E\_p_{i}-E\_p_{i}^{\prime }\right)\;{\;}^{2}}{\left|P\right|}}$$

The accuracy of the base regression models for each machine learning algorithm was evaluated on the test cases (seen in Figure 5) and the whole observed area. Additionally, the execution times were measured for calculating the pixel values within the single image (849 pixels). It should be noted that the measured times include only the prediction phase, whereas the time used for data preparation and the machine learning was excluded. The obtained results are given in Table 3.

**Figure 8:**
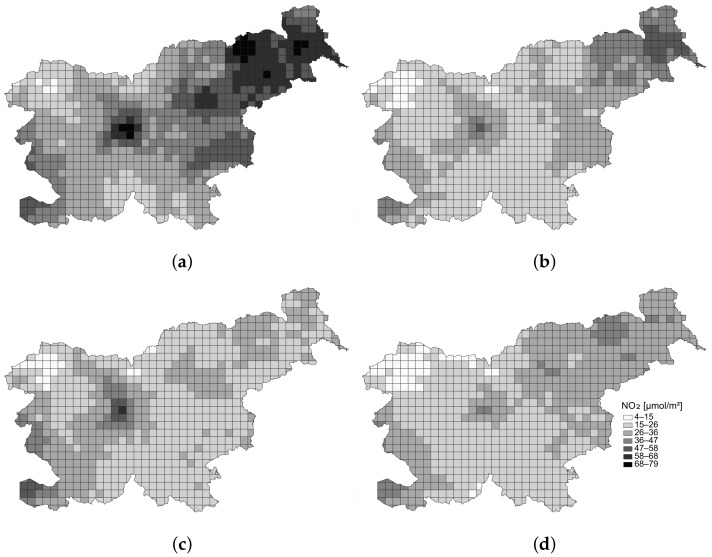
Samples of the satellite-like image, which were calculated by NN in the absence of the satellite data at different time moments: (**a**) UTC 5:00, on 16 March 2021; (**b**) UTC 9:00, on 20 April 2021; (**c**) UTC 17:00, on 24 May 2021; (**d**) UTC 22:00, on 1 June 2021.

As seen in Table 3, the RMSEs varied between test cases. However, the RMSE ratios between them were similar for each base regression model. Likewise, the execution times to calculate the values of pixels in each observed area were also dependent on the regression model. The 1-NN took the longest, while the fastest was LR. The results prove that the method processing time is under 1 min, and that the proposed method is suitable for practical use. However, the time measurement excluded the data preparation and machine learning, which is largely dependent on the size of the training dataset and machine learning algorithm used. Some of the image samples, which were calculated in the absence of the satellite data, are shown in Figure 8.

The additional analyses were conducted on the results obtained by the neural network, as it outperformed the other two algorithms. As seen in Table 4, the RMSE was compared for each test case, based on the number of considered IoT sensors. Some test cases never considered a specific number of IoT sensors. This occurred either due to invalid measurements or positions of the Voronoi cells. These cases are marked with “/” in Table 4 and Table 5.

The accuracy of the algorithm’s performance on test cases also varied because of the distances between them and the considered IoT sensors. This is seen in Table 5, where the average distance was made depending on the number of considered IoT sensors.

Assessment of the NO${}_{2}$ was conducted using additional meteorological variables. They were considered by generating a vector of random meteorological parameters 50 times. In each new iteration the proposed method calculated pixel values for the whole test dataset P. The averages of the results are seen in Table 6, while the best results were obtained when temperature, humidity, wind speed and NO${}_{2}$ were considered together ($RMSE=14.79\times 10^{-6}\;{\mathrm{mol}/m}^{2}$).

## 5. Discussion

A new ensemble approach for the assessment of the arbitrary environmental variable is proposed in this article. It utilizes the data fusion paradigm to construct the satellite-like image, based on measurements from the IoT sensors. Unique to other methods, it omits the interpolation of input variables in the initialization, and performs it during the prediction.

The results showed that, when comparing the method’s performance using three different machine learning algorithms, the feed-forward neural network achieved the best performance. The main factor contributing to the performance of the neural network was its architecture, which determines the number of coefficients used to calculate the target variable. This could have caused us to obtain more accurate results than linear regression. There are also some other differences that contribute to the construction of a regression equation. This includes the number of iterations to update weights, and the algorithm used to determine how the weights are updated.

The RMSEs varied between the selected test cases. The best results on average were obtained in the test cases 5, 7 and 8 (Table 3), which are positioned on the plain terrain (see Figure 5). These cases also had better results when less and nearer sensors were considered (Table 4 and Table 5). This was due to the lack of major geographical barriers between the considered IoT sensors that contributed to the calculated values [15]. Similarly, good results were also obtained in test case 6. This test case is positioned near the Šoštanj Thermal Power Plant and is surrounded by three or more IoT sensors. The test cases with the worst results had geographical barriers present between them and the considered sensors. However, the obtained results could be improved if more IoT sensors were considered.

The 1-NN took the longest time to perform the calculations. This was due to being unsupervised learning, and most processing was performed in the process of searching for the nearest instance. Furthermore, the approach performed slower in cases where the set of pixels with the smallest distance contained many invalid values. In this case, the algorithm had to search for the next set of pixels. The 1-NN also carries out most of the processing in the prediction phase. On the other hand, linear regression and neural network being the supervised approaches, perform most of the calculations in the phase of machine learning.

Nevertheless, it was shown that the proposed approach performs in a relatively short amount of time, and provides good results. The main advantage of this approach is its assessment of the environmental variable at an arbitrary location within the observed area in times when the IoT measurements are available. On the other hand, other similar approaches assess the variables at lower temporal resolutions. The proposed approach increases the spatial resolution by defining the ensemble of base regression models which are dependent on the neighboring IoT sensors. The proposed approach allows the integration of any machine learning algorithm. Furthermore, it can be applied to an arbitrary variable in any observed area, as long as sufficient and appropriate correlated training data are provided. It may also use other auxiliary meteorological variables in addition to the main predictor (NO${}_{2}$ in our case).

The main disadvantage of the proposed approach is its inability to include new sensors. This will be improved by incremental machine learning algorithms [46]. Furthermore, the method performance can also be increased by utilizing different machine learning algorithms to model the relationship between the IoT and the satellite sensor data. We will try to improve efficiency by utilizing recurrent neural networks to make time series predictions [47].

## Figures and Tables

**Table 1:** Querying in archive $A_{\mathrm{IoT}}$ with values of time and E returned by 9 (*N*) sensors. The yellow-colored cells represent valid measurements, which are returned as the result of the query.

Time	$E\_s_{0}$	$E\_s_{1}$	$E\_s_{2}$	$E\_s_{3}$	$E\_s_{4}$	$E\_s_{5}$	$E\_s_{6}$	$E\_s_{7}$	$E\_s_{8}$
$t_{0}$	✗	✓	✓	✓	✗	✗	✓	✓	✗
$t_{1}$	✓	✓	✗	✓	✗	✓	✗	✓	✓
$t_{2}$	✗	✗	✗	✓	✓	✗	✓	✓	✓
$t_{3}$	✗	✓	✓	✓	✗	✗	✓	✗	✗
$t_{4}$	✓	✓	✗	✓	✓	✗	✓	✓	✓
$t_{5}$	✗	✓	✓	✓	✗	✓	✗	✗	✗
$t_{6}$	✗	✓	✗	✓	✓	✓	✓	✓	✓
$t_{7}$	✗	✗	✗	✓	✓	✗	✓	✓	✓

✓: valid measurements, ✗: invalid measurements.

**Table 2:** Querying in archive $A_{\mathrm{Sat}}$ with values of time and E returned by *M* pixels obtained from the satellite images. The yellow-colored cells represent the measurements of satellite pixels, which are returned as the result of the query.

Time	$E\_p_{0}$	$E\_p_{1}$	$E\_p_{2}$	$E\_p_{3}$	$E\_p_{4}$	$E\_p_{5}$	$E\_p_{6}$	$E\_p_{7}$	$E\_p_{8}$	$E\_p_{9}$	$E\_p_{10}$	$E\_p_{11}$	...	$E\_p_{M-1}$
$t_{0}$	✓	✓	✓	✓	✓	✗	✓	✓	✓	✗	✓	✓	…	✗
$t_{1}$	✓	✗	✓	✓	✗	✓	✓	✗	✓	✗	✗	✗	…	✓
$t_{2}$	✓	✓	✓	✓	✓	✓	✓	✗	✓	✓	✓	✗	…	✗
$t_{3}$	✗	✓	✗	✗	✗	✓	✗	✓	✗	✓	✓	✓	…	✗
$t_{4}$	✓	✓	✓	✓	✓	✓	✓	✓	✓	✗	✗	✓	…	✓
$t_{5}$	✗	✓	✓	✗	✓	✓	✗	✓	✓	✓	✓	✓	…	✗
$t_{6}$	✗	✓	✗	✗	✗	✓	✗	✓	✗	✗	✓	✗	…	✗
$t_{7}$	✓	✓	✗	✓	✗	✓	✓	✓	✗	✓	✓	✓	…	✓

✓: valid measurements, ✗: invalid measurements.

**Table 3:** Comparison of RMSE [mol/m${}^{2}$] and execution times on a test dataset, achieved by 1-NN, NN, and LR, on the selected test cases.

BRM	1-NN		LR		NN	
**Area**	**RMSE ($\times 10^{-6}$)**	Execution Times * [s]	**RMSE ($\times 10^{-6}$)**	Execution Time * [μs]	**RMSE ($\times 10^{-6}$)**	Execution Time * [ms]
Test case 1	24.13	1	22.63	7	18.95	2
Test case 2	22.18	2	20.19	6	17.05	3
Test case 3	24.26	2	21.25	7	19.53	4
Test case 4	23.35	6	19.99	7	18.58	3
Test case 5	22.68	1	19.15	7	17.75	2
Test case 6	22.77	12	18.87	8	17.67	4
Test case 7	22.98	7	18.66	7	17.70	1
Test case 8	23.13	4	18.36	6	17.46	1
Whole	21.82	43	16.95	8	15.49	14

BRM: Base Regression Models, *: Rounded, average time measured ×10.

**Table 4:** RMSE [mol/m${}^{2}$] in comparison to the number of considered IoT sensors. The results were obtained from the neural networks, applied on the testing dataset.

Test Case–RMSE ($\times 10^{-6}$)								
Number *	**1**	**2**	**3**	**4**	**5**	**6**	**7**	**8**
3	55.19	/	/	/	/	28.93	/	/
4	16.50	12.08	20.94	/	10.60	17.90	10.57	11.65
5	19.25	10.45	28.72	11.61	18.79	16.81	14.10	18.98
6	12.82	13.94	21.25	15.06	13.93	17.07	18.78	/
7	/	/	20.28	16.70	11.49	17.40	/	/
8	/	2.419	25.48	14.62	/	17.19	/	/
9	/	/	/	/	/	16.66	/	/

*: Number of considered IoT sensors, /: not available.

**Table 5:** The average distances between the IoT sensors and test cases, when different numbers of IoT sensors were considered.

Test Case–Average Distance [km]								
Number *	**1**	**2**	**3**	**4**	**5**	**6**	**7**	**8**
3	60.42	/	/	/	/	1.51	/	/
4	80.89	76.86	23.85	/	44.53	2.95	47.49	60.60
5	81.73	80.96	33.79	28.24	49.24	19.18	47.89	82.24
6	96.07	80.57	38.49	33.55	56.38	17.20	48.18	/
7	/	/	30.91	35.68	62.99	19.81	/	/
8	/	93.71	40.69	38.71	/	44.12	/	/
9	/	/	/	/	/	47.21	/	/

*: Number of considered IoT sensors, /: not available.

**Table 6:** The average of RMSEs [mol/m${}^{2}$] on the testing dataset when each environmental variable was considered.

Considered Variable	Temperature	Wind Speed	Wind Direction	Humidity	${\mathrm{NO}}_{2}$
Test case–RMSE ($\times 10^{-6}$)	16.29	17.27	17.71	17.60	15.47

## Data Availability

Not applicable.
